# Identification of a major *Listeria monocytogenes* outbreak clone linked to soft cheese in Northern Italy – 2009-2011

**DOI:** 10.1186/s12879-017-2441-6

**Published:** 2017-05-12

**Authors:** Ettore Amato, Virginia Filipello, Maria Gori, Sara Lomonaco, Marina Nadia Losio, Antonio Parisi, Pol Huedo, Stephen John Knabel, Mirella Pontello

**Affiliations:** 1 Department of Health Sciences, University of Milan, Via di Rudinì, 8, 20142 Milan, Italy; 2 Department of Veterinary Sciences, University of Turin, Largo P. Braccini, 2, 10095 Grugliasco, Italy; 3 Istituto Zooprofilattico Sperimentale della Lombardia e dell’Emilia Romagna, Via A. Bianchi 9, 25124 Brescia, Italy; 4 Istituto Zooprofilattico Sperimentale della Puglia e della Basilicata, Via Manfredonia 20, 71121 Foggia, Italy; 5 Department of Food Science, The Pennsylvania State University, 405 Rodney A. Erickson Food Science Building, 16802 University Park, State College, PA USA; 6 Coordinated Research Center “EpiSoMI”, University of Milan, Via Carlo Pascal, 36, 20133 Milan, Italy

**Keywords:** *Listeria*, Outbreak clone, Epidemic clone, Molecular methods, Listeriosis

## Abstract

**Background:**

Molecular subtyping and enhanced surveillance in Lombardy region identified a cluster of possibly related listeriosis cases from 2006 to 2010. This cluster grouped 31 isolates that belonged to serotype 1/2a and Sequence Type 38 (ST38) as defined by Multilocus Sequence Typing (MLST).

**Methods:**

Our study expanded the previous investigation to include cases from 2011 to 2014 and used Multi-Virulence-Locus Sequence Typing (MVLST) on all ST38 isolates to better understand their epidemiology and possibly identify a common source outbreak.

**Results:**

Out of 306 *L. monocytogenes* clinical isolates collected, 43 (14.1%) belonged to ST38 with cases occurring in nine out of twelve Lombardy provinces. The ST38 isolates were split by MVLST into two Virulence Types (VTs): VT80 (*n* = 12) and VT104 (*n* = 31). VT104 cases were concentrated between 2009 and 2011 in two provinces, Bergamo and Milan. An epidemiologic investigation was performed and in one case, a matching VT104 isolate was retrieved from a soft cheese sample from a patient’s refrigerator.

**Conclusions:**

Our findings revealed a major listeriosis outbreak in Northern Italy linked to soft cheese in 2009–2011, which went undetected by local health authorities. Our study shows that integrating subtyping methods with conventional epidemiology can help identify the source of *L. monocytogenes* outbreak clones.

**Electronic supplementary material:**

The online version of this article (doi:10.1186/s12879-017-2441-6) contains supplementary material, which is available to authorized users.

## Background

Listeriosis is a foodborne disease caused by *Listeria monocytogenes*, which causes invasive syndromes in people with altered immunity (i.e. immuno-compromised, the elderly, pregnant women, foetuses and the newborns). Symptoms can include severe sepsis or infection of the central nervous system that can lead to lifelong sequelae or death. Pregnancy related listeriosis can result in preterm birth, miscarriage or stillbirth [1, 2]. In healthy people, *L. monocytogenes* may cause a self-limiting febrile gastroenteritis [3].

Within the European Union, notification of listeriosis cases is mandatory. However, in Italy, different notification rates are reported among the various Regions, and to date national coverage is not guaranteed [4]. Moreover, systematic subtyping and comparison with food isolates is fragmented, hindering the investigation and control of possible outbreaks [5]. Lombardy is the most populous region in Italy with 17% of the Italian population [6], and since 2005 it holds an enhanced listeriosis surveillance system that involves a laboratory-based network with voluntary referral of clinical isolates to the Regional Reference Laboratory for foodborne diseases (LabSS). The LabSS collects clinical isolates of *L. monocytogenes* and carries out serotyping and molecular subtyping with Pulsed-Field Gel Electrophoresis (PFGE) and Multilocus Sequence Typing (MLST). This allowed for the period 2006–2014 to report in Lombardy a mean incidence of 0.56 cases/100000 inhabitants in view of the Italian one of 0.20 cases/100000 inhabitants) (data not shown). Between 2006 and 2010, 23% of all collected isolates were identified as Sequence Type 38 (ST38) by MLST, and were grouped in the same PFGE cluster [7]. Given the elevated proportion and the recurrent presence of this ST, there was a possibility these cases were related and belonged to a single outbreak that went undetected by the traditional surveillance system in the Lombardy Region.

Conventional epidemiology typically is inefficient in detecting listeriosis outbreaks as foods with long shelf lives, long incubation periods, and infrequent infections in spite of presumably frequent exposures, may allow a listeriosis outbreak to occur as a succession of apparently unrelated cases [1, 7–9]. Therefore, it is essential that conventional epidemiology be supported by adequate molecular subtyping methods. To date, PFGE is the gold standard for *L. monocytogenes* subtyping, however sequence-based techniques, like MLST, Multi-Virulence-Locus Sequence Typing (MVLST) and core genome MLST [10], have also been successfully applied as they yield unambiguous and highly informative data, which can be easily accessed and exchanged through public databases [11]. MVLST, based on the sequencing of 6 virulence gene fragments, shows excellent epidemiologic concordance [12] (i.e. the ability of a subtyping system to classify epidemiologically related isolates derived from a presumably single-clone outbreak into the same clone [13]); and allows the identification of Epidemic Clones (ECs), strains originated from a single ancestral cell and involved in geographically or temporally unrelated outbreaks [12] .

Our aim was to clarify the epidemiology of listeriosis cases in Lombardy by gaining a deeper insight into ST38 isolates. First, the isolates collection period was expanded by 4 years to include all clinical *L. monocytogenes* isolates up to 2014. Subsequently, all isolates were subtyped using serotyping, PFGE and MLST and those belonging to ST38 were further subtyped using MVLST. These data were analyzed to evaluate the geospatial and temporal distribution of the strains. This study provides an integrated model to better detect and control future listeriosis outbreaks.

## Methods

### Data source and listeriosis case definition

LabSS collected clinical isolates and epidemiologic data. Data are routinely collected using a standardized report form for i) demographic data (e.g. age, gender, province of residence), ii) clinical data (e.g. symptoms, clinical form of disease, existence of underlying conditions, patient outcome) and iii) microbiological data (type and number of the positive biological samples).

For this study, a listeriosis case was defined as isolation of *L. monocytogenes* from a normally sterile body site (e.g. blood or positive cerebrospinal fluid – CSF) or from products of conception. Five food and environmental samples related to the outbreak epidemiologic investigations were retrieved from the Regional Food Safety and Animal Health Institute (Istituto Zooprofilattico Sperimentale della Lombardia ed Emilia Romagna – IZSLER).

### Isolate characterization

All clinical *L. monocytogenes* isolates were identified by standard methods in the clinical microbiology laboratories of healthcare facilities in the Lombardy region. Isolates were either characterized previously [7] or characterized in the current study. Serotyping, PFGE and MLST data were obtained for all isolates, according to the protocol described in the previous work [7]. MVLST was carried out for all those isolates typed as ST38 [7].

#### Serotyping

A multiplex-PCR assay was used to determine the serogroups of *L. monocytogenes* isolates (1/2a-3a; 1/2c-3c; 1/2b-3b; and 4b-4d-4e) [14]. The specific serotype within the serogroups was then determined by conventional serotyping, using *L. monocytogenes* antisera (Denka Seiken Co., Ltd. Tokio, Japan), according to the manufacturer’s instructions.

#### Pulsed-field gel electrophoresis (PFGE)

Isolates obtained up to 2010 were subtyped using PFGE as previously described [7]. For all isolates collected from 2011 to 2014, PFGE was performed according to the PulseNet protocol with the *Asc*I enzyme [15]. Similarity clustering analysis was performed under the same conditions and similarity value (over 80%) as reported in the previous study. Indistinguishable or closely related strains were subsequently restricted with *Apa*I enzyme for cluster confirmation [7].

#### Multilocus sequence typing (MLST) and Multi-virulence-Locus sequence typing (MVLST)

Isolates obtained up to 2010 were subtyped using MLST in the previous study [7]. For all isolates collected from 2011 to 2014, MLST alleles were determined by PCR, using a modified MLST scheme with *ldh* gene and Sanger sequencing as previously described [16]. Alleles and Sequence Types (STs) were assigned by submitting the DNA sequences to the *Listeria* MLST database at the Pasteur Institute, France [17]. MVLST was performed on all isolates belonging to ST38 as previously described [12] by amplification of intragenic regions of six virulence genes (*clpP, dal, inlB, inlC, lisR* and *prfA*). Sequencing was performed by IGA Technology Services (Udine, Italy), using an ABI 3730XL DNA analyzer. Multiple sequence alignments were performed with Molecular Evolutionary Genetic Analysis software (MEGA) v6 [18] and compared with sequences from the *L. monocytogenes* MVLST database [19]. A neighbor-joining tree based on the number of nucleotide differences was constructed using MEGA [18].

### Epidemiologic investigation

Data concerning city of residence, province and date of hospitalization were retrieved from standardized report forms. Epidemiologic interviews conducted by local health authorities were accessed through an official query by the Directorate-General for Health of Lombardy Region. Data were collected from the interviews and foods reported by the patients were divided in categories (meat, cheese, vegetables) in order to identify a potential implicated food.

## Results

### Listeriosis cases

A total of 306 *L. monocytogenes* clinical isolates were analyzed over a 9-year period (2006–2014): 139 from cases occurring in the years 2006–2010 [7] and 167 from cases occurring between the years 2011 and 2014. Overall, 43 (14.1%) out of the 306 *L. monocytogenes* isolates belonged to ST38. Two cases (4.7%) were pregnancy-associated. The mean age of non-pregnancy-associated patients was 59.5 ± 18.0 years, with 51.2% of cases (*n* = 22) observed in people older than 70 years. *L. monocytogenes* was isolated from blood (*n* = 32) and from CSF (*n* = 6). Two cases allowed isolation of *L. monocytogenes* from both blood and CSF, one case from placenta, and no information was available in 2 cases. Cases with underlying medical conditions were 35 (81%) out of 39. The all-cause fatality rate of 24 cases with a known outcome was 28%. Cases due to ST38 peaked in 2009, 2010 and 2011 (*n* = 10, 16 and 10 cases respectively), while this *L. monocytogenes* subtype was observed sporadically in 2006, 2008 and 2014 (2 cases/year each), and in 2013 (*n* = 1). Cases occurred in nine out of twelve Lombardy provinces, with the highest frequencies observed in the provinces of Bergamo and Milan.

### Isolate characterization

All 306 clinical isolates were serotyped and analysed by PFGE and MLST. The most prevalent serotypes were 1/2a (*n* = 182, 59.5%) and 4b (*n* = 75, 24.5%), accounting for 84%of the total isolates. Thirteen PFGE clusters were identified, including twelve clusters previously reported [7] and a new cluster (Cluster 13) related to the 2011–2014 period. In the previous study [7], PFGE clustering overall correlated with MLST clustering, although two PFGE clusters (Cluster 2 and 3) belonged to the same ST (ST1). The most prevalent STs were ST38 (*n* = 43, 14.1%), ST1 (*n* = 37, 12.1%), ST8 (*n* = 27, 8.8%), ST155 (*n* = 22, 7.2%), ST2 (*n* = 17, 5.6%), and ST3 (*n* = 17, 5.6%). These six STs accounted for 39.3% of the total *L. monocytogenes* clinical isolates collected during the period.

#### In-depth analysis of isolates belonging to ST38

**Fig. 1:**
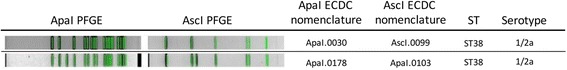
Pulsed field gel electrophoresis profiles of the *L. monocytogenes* isolates identified as ST38 with official *Asc*I-*Apa*I pulsotype codes as provided by The European Surveillance System

All isolates identified as ST38 belonged to serotype 1/2a and were grouped in Cluster 11 by PFGE. Isolates in this cluster showed two PFGE profiles, identified as *Asc*I.0099-*Apa*I.0030 and *Asc*I.0103-*Apa*I.0178 (Fig. 1). The European Surveillance System (TESSy) officially provided the *Asc*I-*Apa*I pulsotype codes. These two PFGE profiles shared a high degree of similarity (89.9%) and differed by one band using *AscI* and two bands using *Apa*I. No similarity was observed when they were compared with the profiles in the TESSy database (ECDC, Stockholm).

**Fig. 2:**
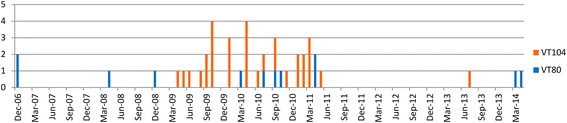
Temporal distribution of the 43 ST38 *L. monocytogenes* clinical isolates collected in Lombardy (2006–2014) according to the two different Virulence Types (VT80 and VT104)

**Fig. 3:**
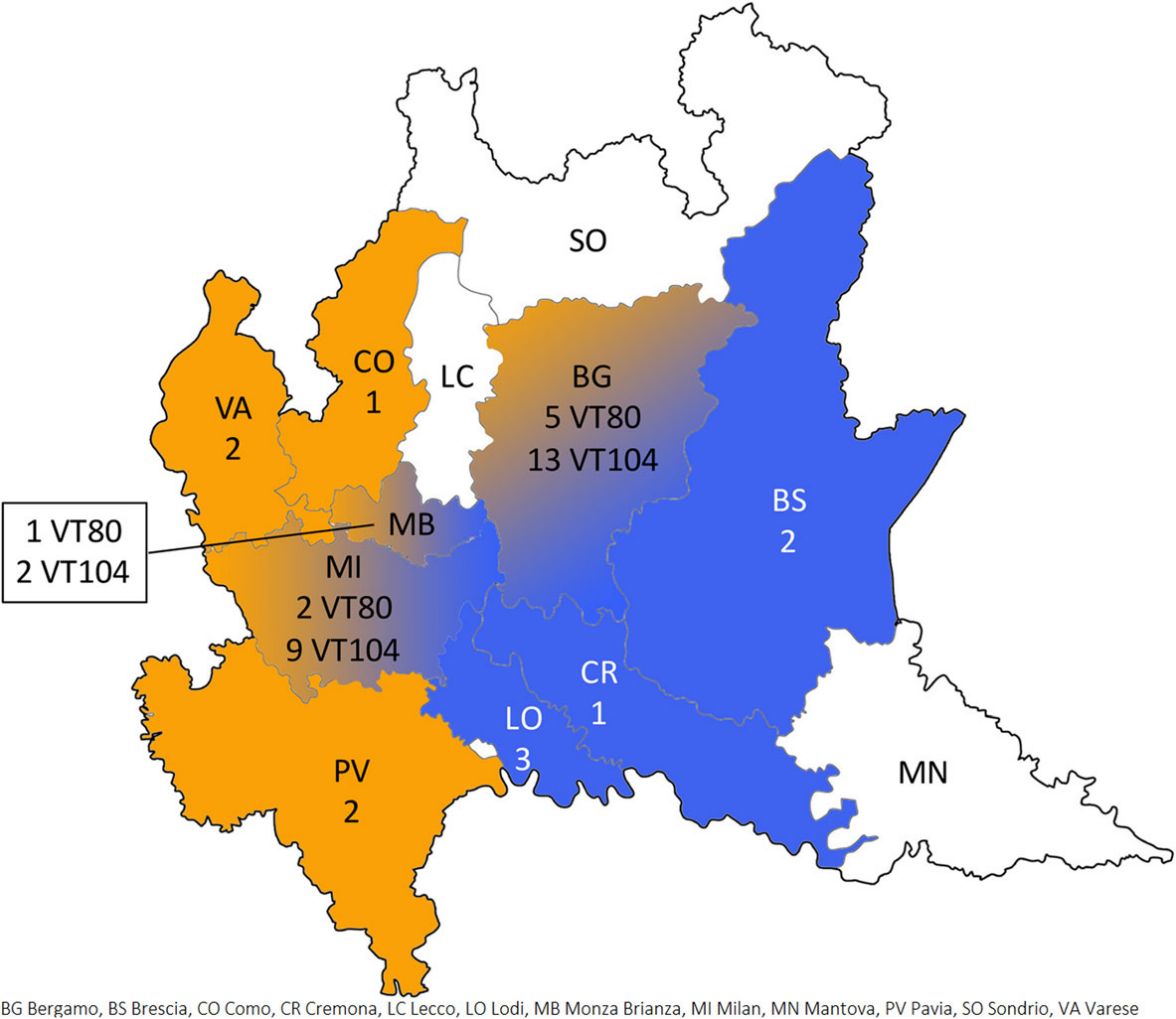
Spatial distribution of the 43 ST38 listeriosis cases in Lombardy (2006–2014). Orange provinces indicate VT80 cases, blue provinces indicate VT104 cases, while gradient provinces indicate both VT80 and VT104 cases

MVLST split the 43 ST38 isolates into two Virulence Types (VTs): VT80 (*n* = 12) and VT104 (*n* = 31), which differed by a Single Nucleotide Polymorphism (SNP) at the 90th position in *dal*. These VTs had different spatiotemporal distributions, with VT80 being sporadically observed from 2006 to 2014 in the Western and Central provinces of Bergamo, Varese, Pavia, Milan, Como and Monza Brianza provinces, and VT104 being concentrated between 2009 and 2011in the Eastern and Central provinces of Bergamo, Milan, Lodi, Monza Brianza, Brescia and Cremona (Figs. 2 and 3). The province with the highest annual incidence for ST38 was Bergamo (0.19 cases/100,000 inhabitants) followed by Lodi (0.15 cases/100,000 inhabitants). For VT80 Bergamo province had the highest incidence (0.05cases/ 100,000 inhabitants), followed by Pavia province (0.04 cases/100,000 inhabitants). While, for VT104 the highest incidence was in Lodi province (0.15 cases/100,000 inhabitants) followed by Bergamo (0.14cases/100,000 inhabitants) (Table 1).

**Table 1:** Mean annual listeriosis incidence (cases/100,000 inhabitants) in Lombardy by province^a^ for ST38 (*n* = 43), VT80 (*n* = 12) and VT104 (*n* = 31) over the period 2006–2014

**ST/VT**	**BG**	**BS**	**CO**	**CR**	**LC**	**LO**	**MB**	**MN**	**MI**	**PV**	**SO**	**VA**	**Lombardy**
	(1.02)	(0.52)	(0.44)	(0.85)	(1.00)	(0.71)	(0.55)	(0.22)	(0.70)	(0.44)	(1.10)	(0.50)	(0.56)
ST38	**0.19**	0.02	0.02	0.03	0	**0.15**	0.04	0	0.04	0.04	0	0.03	0.05
VT80	**0.05**	0	0.02	0	0	0	0.01	0	0.01	**0.04**	0	0.03	0.02
VT104	**0.14**	0.02	0	0.03	0	**0.15**	0.03	0	0.03	0	0	0	0.03

In brackets the mean incidence for all listeriosis cases (*n* = 306)Values in bold are the provincial incidences above the mean annual regional incidence

^a^BG – Bergamo, BS – Brescia, CO – Como, CR – Cremona, LC – Lecco, LO – Lodi, MB – Monza Brianza, MI – Milan, MN – Mantova, PV – Pavia, SO – Sondrio, VA – Varese

### Epidemiologic investigation

Local health authorities carried out epidemiologic interviews in 13 out of the 43 cases. Three food categories were listed by the interviewed patients: cheese (all patients), vegetables (*n* = 5, 38.5%) and meat (*n* = 3, 23.1%). In particular, 4 patients reported the consumption of smear-ripened cheese, namely Protected Designation of Origin (PDO) Taleggio cheese. For one patient (corresponding to isolate LMO122; Additional file 1: Table S1) that lived in Bergamo province, one sample of Taleggio cheese was collected from the household refrigerator and tested positive for *L. monocytogenes* (isolate LMO129; Additional file 1: Table S1). Following this finding the local health authorities traced back the production plant (Plant A) of the implicated PDO Taleggio cheese, located in the Bergamo province, and performed an environmental sampling that led to the isolation of *L. monocytogenes* (isolate LMO130; Additional file 1: Table S1). Both the cheese and the environmental isolates shared the same PFGE profile as the clinical isolate (Cluster 11 as defined by Mammina et al. [7]). The correlation was further confirmed by MVLST that identified LMO122, LMO129 and LMO130 as belonging to VT104. The PFGE *L. monocytogenes* database of IZSLER was queried against Cluster 11 pulsotypes in order to identify food samples sharing the same molecular profile. Three isolates sharing the same pulsotype and originating from 2 different plants were found (IZSA-C, Additional file 1: Table S1). In particular, IZSC was from a sample of Taleggio cheese produced in Plant A, and IZSA and IZSB were from two samples of Taleggio cheese produced in a different plant (Plant B) also located in the Bergamo province. These three cheese isolates were subsequently typed with MVLST and identified as VT104 (IZSC) and VT80 (IZSA-B).

The *Listeria* MLST database, was consulted to verify the isolation of ST38 in other food sources. It was found that ST38 has only three records of isolates from clinical cases (France, 1997, Oceania, 2009 and Germany, 2011), and no records of food samples [17]. Similarly, only two ST38 isolates are listed in the MLST Italian database at the IZS of Apulia and Basilicata, one of which had been isolated in 2005 from a sample of PDO Gorgonzola cheese [7, Parisi A, personal communication; 2016].

## Discussion

Based on temporal and epidemiologic data, we hypothesize that strains belonging to the previously detected Cluster 11 [7] were part of an outbreak that went undetected by the traditional surveillance system. Our findings reveal that between 2006 and 2014, isolates belonging to ST38 represented the most prevalent *L. monocytogenes* subtype detected in Lombardy. However, the overall mean age and case fatality rate of ST38 cases were comparable to those previously reported [7]. Notably, the presence of ST38 strains may have played a role in finding in a previous study serotype 1/2a more prevalent than serotype 4b in Lombardy [7]. This trend is consistent with the worldwide scenario, where serotype 1/2a is now competing with 4b as the leading serotype responsible for clinical cases [20, 21].

The observed peak of cases (2009 and 2011), identified by all subtyping methods used, is consistent with the occurrence of an outbreak rather than a surveillance artifact. In fact, the plotting of cases over time yields a typical epidemic curve and the presence of an epidemic event was confirmed by spatiotemporal analysis of the distribution of cases belonging to ST38 using a mathematical model [22]. Moreover, in Bergamo – the province with the highest number of cases – such epidemic event may have caused the increase of listeriosis incidence for the years 2010 and 2011 as registered by the notification system.

MVLST divided the ST38 isolates in two VTs (VT80 and VT104), characterized by a different spatiotemporal distribution, where the peak in the number of cases between 2009 and 2011 is mainly due to VT104 (Figs. 2 and 3). The two VTs differ only in the 90th nucleotide of the *dal* gene fragment analyzed, which is a synonymous mutation. The first VT80 clinical cases originated in Bergamo province in 2006, while the first cases of VT104 were reported in 2009 especially in the Eastern provinces of Lombardy (i.e. Cremona, Lodi, Milan and Bergamo) (Fig. 3).

We can reasonably hypothesize that Taleggio cheese was the implicated food, based on the following considerations: i) all patients reported cheese as suspected food and four specifically recalled consumption of Taleggio cheese, ii) a direct correlation between a clinical case and the Taleggio cheese sample retrieved from the patient’s refrigerator was confirmed by PFGE and MVLST, iii) all routinely collected food isolates with the same pulsotypes and VTs of those in the ST38 cluster are from Taleggio cheese samples, and iv) the molecular type found (ST38, Cluster 11 pulsotypes [7], VT104) is infrequent and never reported before in other food sources [17, 19]. Based on these assumptions and to the best of our knowledge, we can designate VT104 as the first outbreak clone detected in Italy.

Typically, many *L. monocytogenes* outbreaks originate from food processing plants that are colonized by the outbreak clone, which cause the contamination of the final product [23]. In this light, PDO Taleggio producing plants were identified as the hypothetical source: VT104 was found in a production plant (Plant A) in Bergamo province following the sampling relative to the epidemiologic investigation linked to a patient; while VT80 was found in a different PDO Taleggio production plant (Plant B), also located in the Bergamo province, during routine monitoring carried out by IZSLER.

We can hypothesize, as supported by the timeline (Fig. 2), that VT80 may have first appeared in one plant in the Bergamo province and caused an undetected outbreak (Figs. 2 and 3, *orange*). Then either in the same plant or after being transferred to a second one, in the same province (Bergamo), it evolved into VT104 and caused a second outbreak detected herein (Fig. 3, *blue*). The two VTs have different but overlapping distribution in Western and Eastern Lombardy, likely due to the fact that the processing plant colonized with VT80 may have also shipped contaminated product to the East part of Lombardy and the processing plant colonized with VT104 also shipped product to the West. It is interesting to note that no VT80 or VT104 cases have been reported in Lecco and Sondrio provinces (Fig. 3, Table 1), even though these provinces showed the highest listeriosis incidences together with Bergamo (Table 1) and have a similar dairy production. This may be explained by the fact that in Italy dairy products have a strong artisanal and local connotation that may limit the geographic diffusion of *L. monocytogenes* strains. Nevertheless, considering that many Italian local products are also exported, it may be possible that a contaminated dairy product will be responsible for a listeriosis case abroad while not cause any case in a neighboring producing area.

Interestingly, the PFGE profiles of isolates in Cluster11 were closely related to the pulsotype (*Asc*I.0087) of one clinical case involved in the 2012 US outbreak linked to ricotta salata produced in Southern Italy and of two clinical cases detected in Lombardy in 2011 [24]. The isolates linked to the ricotta salata outbreak were typed as VT80 [19, 25], while the two Italian cases were subtyped as ST101, which is grouped in the same Clonal Complex (CC) as ST38 (i.e. CC101). On the other hand, VT80 has also been found in other cheeses as PDO Taleggio and Gorgonzola cheese, two products that have an overlapping producing area (limited exclusively to Lombardy and Piedmont) [19, 26]. These findings suggest that this molecular type (VT80, corresponding to ST38 and ST101, both belonging to CC101) might be common in dairy production facilities in Italy, particularly those manufacturing cheese. Haase et al. [27] reported that CC101 isolates were frequently isolated from clinical cases in the mid-1950s, but only rarely in recent times, and this may have hampered the designation of a correlated EC. For instance, it is still unknown if the ricotta salata and the taleggio outbreak have some kind of connection, but we can hypothesize the involvement of a common mean of transmission between dairy processing facilities, such as milk and/or transportation vehicles. Therefore, considering that the definition of an EC is a strain or group of strains originated from a single ancestral cell and involved in geographically or temporally unrelated outbreaks [12], it can be argued that the Taleggio outbreak clone (CC101/ST38/VT104) and the ricotta salata outbreak clone (CC101/ST101/VT80) can be considered as a novel EC, namely ECXI.

Unfortunately, the epidemiologic interviews were available only in 13 out of the 43 cases (with 11 cases belonging to VT104 and two to VT80). This shortage of information limited the possibility of identifying with more certainty the food vehicle associated to VT80, which in turn could have disclosed possible connections with the ricotta salata outbreak.

However, this study suggests how the application of sequence based typing methods can support the rapid detection and control of outbreak events, especially when cases do not immediately appear correlated (e.g. they are spread in time and space). Also, this study highlights the importance of public typing databases, pivotal in supporting epidemiological investigations. Moreover, the specific type found (ST38 or VT80/104) had only been rarely reported before and it appears now to have adapted to the dairy production environmental niche. Therefore, the results of this study could be useful in the investigation of other outbreaks linked to this molecular type and in the monitoring of strain persistence in processing plants.

## Conclusions

Our findings revealed a major outbreak in Northern Italy linked to PDO Taleggio cheese in 2009–2011, which went undetected by local health authorities. The closely related outbreak clones causing this outbreak represent a novel epidemic clone (ECXI) that was also linked to the U.S. ricotta salata outbreak. Our results suggest that traditional molecular subtyping methods (e.g. PFGE) should be integrated with DNA sequence-based methods (e.g. MLST and MVLST) and space-time analysis models, in order to rapidly detect and control outbreaks that otherwise would have been neglected [28].

## Additional file

Additional file 1: Table S1. Isolate and subtyping data of the 48 analyzed *L. monocytogenes* isolates collected from 2006 to 2014 in the Lombardy Region in Italy from clinical (*n* = 43), food (*n* = 4), and environmental (*n* = 1) sources. All clinical isolates were previously serotyped as 1/2a and typed as sequence type 38 (ST38), which belongs to clonal complex 101 (CC101). All isolates share the pulsotypes identified in Cluster 11 in Mammina et al. [7]. ^a^BG – Bergamo, BS – Brescia, CO – Como, CR – Cremona, LC – Lecco, LO – Lodi, MB – Monza Brianza, MI – Milan, MN – Mantova, PV – Pavia, SO – Sondrio, VA – Varese. ^b^CSF: cerebrospinal fluid. ^c^VT: Virulence Type. (DOCX 18 kb)

## Abbreviations

CC: Clonal Complex
CSF: cerebrospinal fluid
EC: Epidemic Clone
IZSLER: IstitutoZooprofilatticoSperimentaledellaLombardiaed Emilia Romagna
LabSS: Regional Reference Laboratory for foodborne diseases
MLST: Multilocus Sequence Typing
MVLST: Multi-Virulence-Locus Sequence Typing
PDO: Protected Designation of Origin
PFGE: Pulsed-Field Gel Electrophoresis
ST: Sequence Type
VT: Virulence Type
