# Supplementary material for: Identification of a major Listeria monocytogenes outbreak clone linked to soft cheese in Northern Italy – 2009-2011
Source: BMC Infect Dis. 2017 May 12;17:342. doi: 10.1186/s12879-017-2441-6 (PMC5429568; doi:10.1186/s12879-017-2441-6)
Supplement: Additional file 1: Table S1. — Isolate and subtyping data of the 48 analyzed L. monocytogenes isolates collected from 2006 to 2014 in the Lombardy Region in Italy from clinical (n = 43), food (n = 4), and environmental (n = 1) sources. All clinical isolates were previously serotyped as 1/2a and typed as sequence type 38 (ST38), which belongs to clonal complex 101 (CC101). All isolates share the pulsotypes identified in Cluster 11 in Mammina et al. [7]. aBG – Bergamo, BS – Brescia, CO – Como, CR – Cremona, LC – Lecco, LO – Lodi, MB – Monza Brianza, MI – Milan, MN – Mantova, PV – Pavia, SO – Sondrio, VA – Varese. bCSF: cerebrospinal fluid. cVT: Virulence Type. (DOCX 18 kb) [file 12879_2017_2441_MOESM1_ESM.docx]

| **Isolate** | **Year** | **Province^a^** | | **Source** | **Site of isolation^b^** | **VT^c^** |
| --- | --- | --- | --- | --- | --- | --- |
| LMO 17 | 2006 | BG | Clinical | | CSF | 80 |
| LMO 18 | 2006 | BG | Clinical | | Blood | 80 |
| LMO 50 | 2008 | VA | Clinical | | Blood | 80 |
| LMO 64 | 2008 | BG | Clinical | | CSF | 80 |
| LMO 77 | 2009 | CR | Clinical | | Blood | 104 |
| LMO 104 | 2009 | BG | Clinical | | Blood | 104 |
| LMO 105 | 2009 | BG | Clinical | | CSF - Blood | 104 |
| LMO 106 | 2009 | MB | Clinical | | Blood | 104 |
| LMO 107 | 2009 | MB | Clinical | | Blood | 104 |
| LMO 109 | 2009 | MI | Clinical | | Blood | 104 |
| LMO 110 | 2009 | MI | Clinical | | unknown | 104 |
| LMO 114 | 2009 | LO | Clinical | | Blood | 104 |
| LMO 115 | 2009 | LO | Clinical | | Blood | 104 |
| LMO 111 | 2010 | MI | Clinical | | Blood | 104 |
| LMO 112 | 2010 | BG | Clinical | | Blood | 104 |
| LMO 119 | 2010 | BG | Clinical | | Blood | 104 |
| LMO 120 | 2010 | BG | Clinical | | CSF | 80 |
| LMO 122 | 2010 | BG | Clinical | | CSF | 104 |
| LMO 123 | 2010 | MI | Clinical | | Blood | 104 |
| LMO 125 | 2010 | MI | Clinical | | Blood | 104 |
| LMO 127 | 2010 | MI | Clinical | | Blood | 104 |
| LMO 134 | 2010 | MI | Clinical | | CSF - Blood | 104 |
| LMO 138 | 2010 | BS | Clinical | | Blood | 104 |
| LMO 141 | 2010 | BG | Clinical | | Placenta | 80 |
| LMO 145 | 2010 | BG | Clinical | | Blood | 104 |
| LMO 147 | 2010 | BG | Clinical | | Blood | 104 |
| LMO 152 | 2010 | VA | Clinical | | Blood | 80 |
| LMO 159 | 2010 | LO | Clinical | | Blood | 104 |
| LMO 160 | 2010 | MI | Clinical | | Blood | 80 |
| LMO 164 | 2011 | MI | Clinical | | Blood | 104 |
| LMO 166 | 2011 | BG | Clinical | | Blood | 104 |
| LMO 168 | 2011 | MI | Clinical | | Blood | 104 |
| LMO 171 | 2011 | BG | Clinical | | Blood | 104 |
| LMO 172 | 2011 | BG | Clinical | | Blood | 104 |
| LMO 175 | 2011 | BG | Clinical | | CSF | 104 |
| LMO 177 | 2011 | MI | Clinical | | Blood | 104 |
| LMO 179 | 2011 | MI | Clinical | | Blood | 104 |
| LMO 183 | 2011 | PV | Clinical | | Blood | 80 |
| LMO 221 | 2011 | PV | Clinical | | Blood | 80 |
| LMO 253 | 2013 | BG | Clinical | | Blood | 104 |
| LMO 266 | 2009 | BS | Clinical | | CSF | 104 |
| LMO 296 | 2014 | CO | Clinical | | Blood | 80 |
| LMO 302 | 2014 | MI | Clinical | | unknown | 80 |
| LMO 129 | 2010 | BG | Food | | Taleggio cheese A | 104 |
| LMO 130 | 2010 | BG | Environmental | | Production plant A | 104 |
| IZSA | 2011 | BG | Food | | Taleggio cheese B | 80 |
| IZSB | 2011 | BG | Food | | Taleggio cheese B | 80 |
| IZSC | 2011 | BG | Food | | Taleggio cheese A | 104 |

**Table S1 –** Isolate and subtyping data of the 48 analyzed *L. monocytogenes* isolates collected from 2006-2014 in the Lombardy Region in Italy from clinical (n= 43), food (n=4), and environmental (n=1) sources. All clinical isolates were previously serotyped as 1/2a and typed as sequence type 38 (ST38), which belongs to clonal complex 101 (CC101). All isolates share the pulsotypes identified in Cluster 11 in Mammina et al. [7]. ^a^BG – Bergamo, BS – Brescia, CO – Como, CR – Cremona, LC – Lecco, LO – Lodi, MB – Monza Brianza, MI – Milan, MN – Mantova, PV – Pavia, SO – Sondrio, VA – Varese. ^b^CSF: cerebrospinal fluid. ^c^VT: Virulence Type.
